# Dynamic control of light emission faster than the lifetime limit using VO_2_ phase-change

**DOI:** 10.1038/ncomms9636

**Published:** 2015-10-22

**Authors:** Sébastien Cueff, Dongfang Li, You Zhou, Franklin J. Wong, Jonathan A. Kurvits, Shriram Ramanathan, Rashid Zia

**Affiliations:** 1 School of Engineering and Department of Physics, Brown University, Providence, Rhode Island 02912, USA; 2 School of Engineering and Applied Sciences, Harvard University, Cambridge, Massachusetts 02138, USA

## Abstract

Modulation is a cornerstone of optical communication, and as such, governs the overall speed of data transmission. Currently, the two main strategies for modulating light are direct modulation of the excited emitter population (for example, using semiconductor lasers) and external optical modulation (for example, using Mach–Zehnder interferometers or ring resonators). However, recent advances in nanophotonics offer an alternative approach to control spontaneous emission through modifications to the local density of optical states. Here, by leveraging the phase-change of a vanadium dioxide nanolayer, we demonstrate broadband all-optical direct modulation of 1.5 μm emission from trivalent erbium ions more than three orders of magnitude faster than their excited state lifetime. This proof-of-concept demonstration shows how integration with phase-change materials can transform widespread phosphorescent materials into high-speed optical sources that can be integrated in monolithic nanoscale devices for both free-space and on-chip communication.

## Abstract (web summary)

Erbium ions offer a way to integrate light emitters into silicon electronics, but their radiative decay time is too slow for effective light modulation. Here, the authors use phase changes in vanadium dioxide to enable all-optical modulation more than a thousand times faster than the erbium excited-state lifetime.

## Graphical abstract

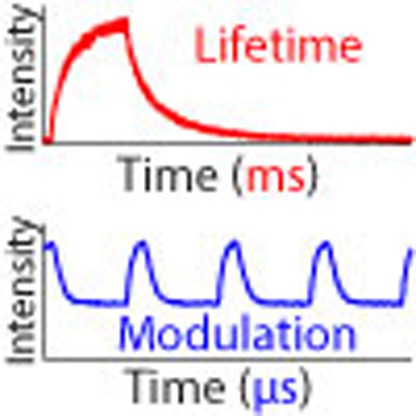

The tunable properties of phase-change materials provide an exciting opportunity to modulate the optical response of optoelectronic devices, potentially at ultrafast speeds^1^,^2^,^3^,^4^. For example, phase-change materials have been considered as a means to modulate reflection and transmission of nanoscale films and devices^5^,^6^,^7^,^8^. We propose here a new framework to directly modulate light emission from integrated sources faster than their radiative lifetime. The concept is based on the dynamic manipulation of light through tailoring the local density of optical states (LDOS). Such LDOS engineering can be used to control the direction^9^, polarization^10^,^11^ and spectrum^12^,^13^ of light emission, even at sub-lifetime scales^14^.

Here, we use phase-change materials within an engineered optical environment to dynamically control the spontaneous light emission of a quantum emitter. We leverage the ultrafast insulator-to-metal transition (IMT) of vanadium dioxide (VO_2_)^2^,^3^,^4^ as well as the symmetry difference in the polarization of electric dipole (ED) and magnetic dipole (MD) transitions of erbium ions to demonstrate direct all-optical modulation of spontaneous emission at sub-lifetime scales. Distinct from direct ‘electronic' modulation of off-chip III-V based lasers^15^,^16^,^17^ or optical modulation using external interferometers^18^,^19^,^20^, this approach enables both the emitter and modulator to be monolithically integrated into a single nanoscale device.

## Results

### Design

**Figure 1:**
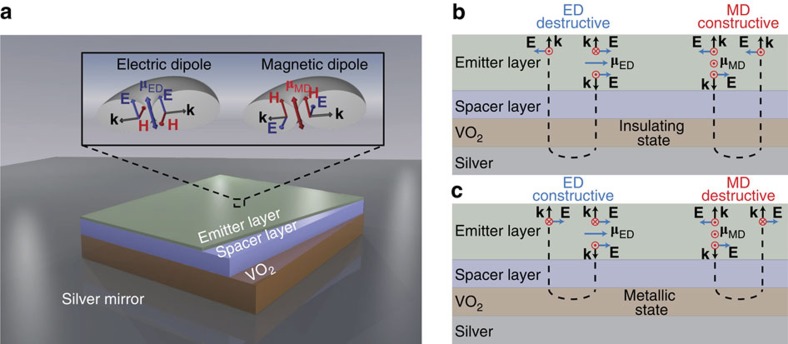
Sketch of the sample and illustration of LDOS modulation. (**a**) Sketch of the sample. Inset shows the polarization symmetries of ED and MD radiation. (**b**,**c**) illustrate the interference of ED and MD emission processes when the VO_2_ layer is in the insulating and metallic phases, respectively.

The key to realizing optical modulation is to design a multilayer structure such that the phase-change layer can be externally switched by a control laser while also having maximum influence on the LDOS of the emitter layer. A simple design to achieve this goal is a quarter-wavelength phase-change layer (that is, thickness 
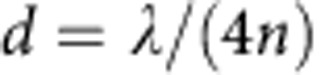
 where *n* is the refractive index) located between an emitter layer and a metal mirror, as shown in Fig. 1a. If the stack is constructed in this way, there is a 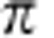 phase shift in the effective optical path length when the VO_2_ is switched from the insulating to metallic state (Fig. 1b,c), which maximizes the influence of the phase-change on the surrounding LDOS.

**Figure 2:**
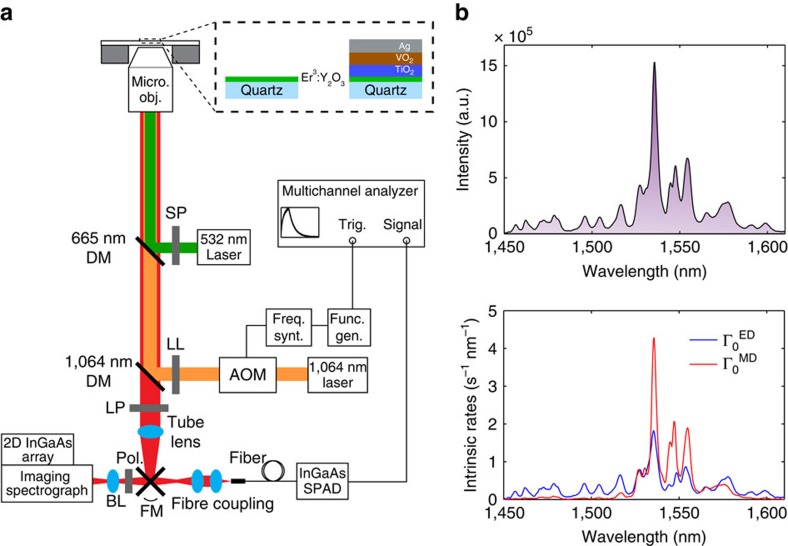
Experimental set-up and ED/MD contributions to Er^3+^ emission around 1.5 μm. (**a**) Experimental set-up used for conventional photoluminescence, energy-momentum spectroscopy and time-resolved measurements. (**b**) Photoluminescence spectrum of a single-layer Er^3+^:Y_2_O_3_ sample. (**c**) Extracted spectrally resolved intrinsic emission rates for ED (blue) and MD (red) transitions obtained from theoretical fit to experimental energy-momentum spectra (Supplementary Fig. 1). AOM, acousto-optic modulator; BL, Bertrand lens; DM, dichroic mirror; FM, flip mirror; freq. synt., frequency synthesizer; func. gen., function generator; LL, laser line; micro. obj., microscope objective; Pol, polarization; SP, short-pass; SPAD, single-photon avalanche photodiode; trig., trigger.

As the process of light emission depends both on the optical environment and on the intrinsic properties of the emitter, engineering spontaneous emission also requires knowledge of the underlying electronic transitions (that is, emission wavelength and dipolar nature). The emission from erbium at 1.5 μm has a multipolar character, showing equal contributions from ED and MD transitions^13^,^21^. Using the energy-momentum spectroscopy set-up shown in Fig. 2a, we can precisely quantify the intrinsic radiative rates of ED and MD transitions in a thin film of Er^3+^:Y_2_O_3_ (refs 13, 22; Supplementary Fig. 1). The results of this analysis illustrate how the photoluminescence spectrum (shown in Fig. 2b) originates from a combination of spectrally distinct ED and MD transitions (shown in Fig. 2c)^13^. These two kinds of transitions typically present differing field symmetries: ED emit with symmetric electric fields, while MD emit with antisymmetric electric fields (Fig. 1c). This symmetry difference can be leveraged to dynamically enhance (or suppress) their emission by modifying the electric and magnetic LDOS via the VO_2_ IMT and therefore switch between these distinct spectra.

**Figure 3:**
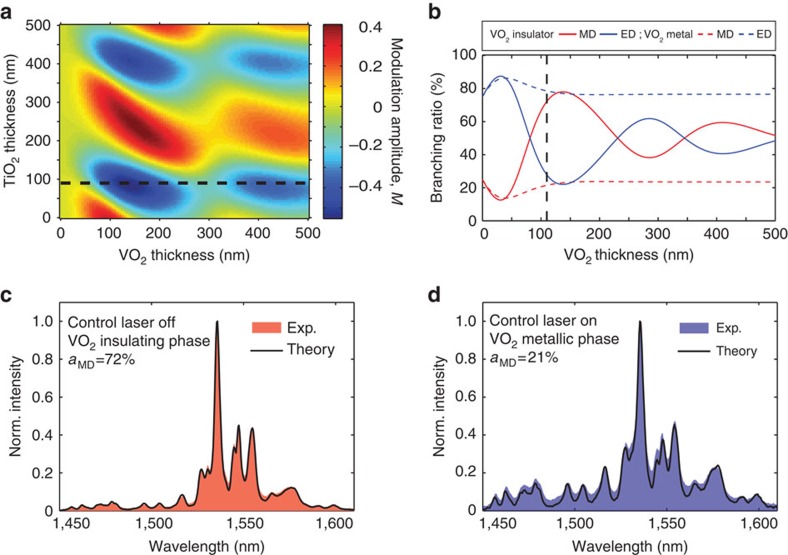
Device design and spectral modulation of Er^3+^ emission in multilayer structure. (**a**) 2D colour plot of the calculated modulation amplitude of the MD contribution to Er^3+^ emission at 1.5 μm upon the VO_2_ phase-change, as a function of TiO_2_ and VO_2_ thicknesses. (**b**) Evolution of the branching ratio of ED (red) and MD (blue) emission as a function of the VO_2_ thickness (where 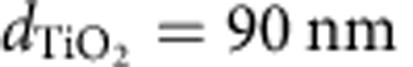 as indicated by the black line in **a**), before (solid lines) and after (dashed lines) inducing the VO_2_ insulator-to-metal transition. The black line labels the VO_2_ thickness used in our experiment. (**c**,**d**) Experimental and calculated spectra for Er^3+^ ions when VO_2_ is in the (**c**) insulating state and (**d**) metallic state. Experimental spectra are shown in shaded red and blue colour, respectively, whereas theoretically predicted spectra are shown as black solid lines. Exp, experimental; norm, normalized.

To this end, we design a multilayer structure (Fig. 1) that comprises an Er^3+^:Y_2_O_3_ thin-film emitter, a TiO_2_ spacer layer, a VO_2_ layer and an Ag mirror. (Expressions for the LDOS in such a five-layer system are explicitly provided in Supplementary Note 1 together with a related schematic in Supplementary Fig. 2). Figure 3a shows the calculated modulation amplitude of MD emission as a function of the thicknesses of both VO_2_ and TiO_2_ layers, where 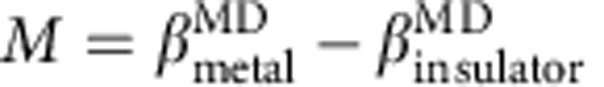 and 
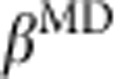
 denotes the Er^3+^ MD branching ratio for the different phases of VO_2_ (more details in Methods section).

### Switching ED and MD emission using VO_2_

As can be seen in Fig. 3a, specific conditions enable the complete reversal of the emission from dominantly MD to dominantly ED (and vice-versa) upon switching of VO_2_. To experimentally investigate this direct modulation of Er^3+^ light emission induced by the VO_2_ phase-change, we fabricated a multilayer structure with 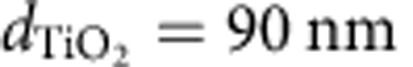 and 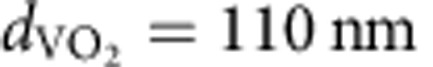. For this particular geometry, the device is designed such that the emitter layer has a high magnetic LDOS when VO_2_ is in the insulating state, but switches to a high electric LDOS when VO_2_ is in the metallic state as shown in Fig. 3b.

**Figure 4:**
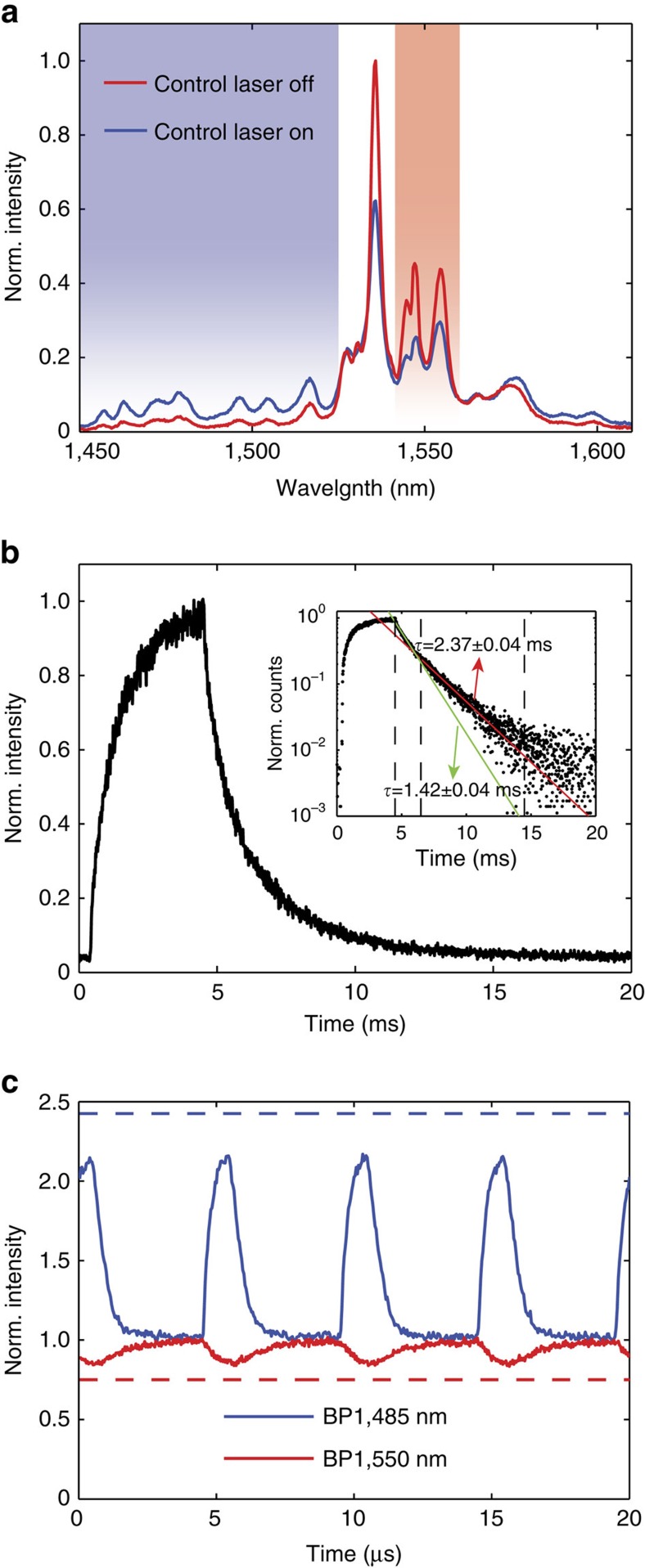
Sub-lifetime modulation of Er^3+^ light emission. (**a**) Experimental spectra for Er^3+^ ions when VO_2_ is in the insulating state (red) and in the metallic state (blue). (**b**) Decay trace of the time-resolved photoluminescence intensity from Er^3+^ ions subjected to a pulsed 532 nm excitation. Inset shows lifetime fits, showing both fast (green) and slow (red) decay contributions with lifetimes of 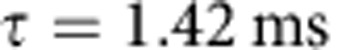 and 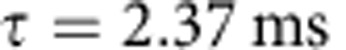, respectively. (**c**) Time-resolved normalized photoluminescence of the Er^3+^ ions in the multilayer structure when continuously pumped by a 532-nm laser while VO_2_ is simultaneously switched by a pulsed 1,064 nm excitation at a 200 kHz repetition rate. Note the three orders of magnitude difference in the time-scale for **c** as compared with **b**. Blue and red solid lines represent two different spectral ranges of the bandpass (BP) filters (1,450–1,520 nm and 1,538–1,562 nm, respectively). The dashed lines show the theoretical maximum modulation calculated according to the spectra in **a**.

The shaded curve in Fig. 3c shows the measured spectrum of the sample when continuously pumped by a 532 nm laser. As expected, the spectrum resembles that of dominantly MD emission (as can be seen by comparison to the intrinsic MD rate in Fig. 2c). Then, using a 1,064 nm control beam, we independently trigger the VO_2_ phase-change and demonstrate all-optical modulation of the local optical environment as well as the resulting emission. (Note that the 1,064 nm control laser is chosen to be non-resonant with any Er^3+^ transitions). Figure 3d shows the resulting Er^3+^ emission spectrum under illumination by both the 1,064 nm control laser and 532 nm pump laser and we see a clear difference in the resulting spectrum. As compared with Fig. 3c, the spectra in Fig. 3d shows the rise of distinct emission peaks between 1,450 and 1,520 nm, which indicates a switch from dominantly MD emission to dominantly ED emission (more details in the Supplementary Fig. 3). This is more clearly evidenced in Fig. 4a where the experimental Er^3+^ spectra obtained for insulating and metallic VO_2_ are displayed together. In addition, when the 1,064 nm laser control beam is turned back off, the Er^3+^ emission returns to its initial spectral shape.

From the measured intrinsic rates (Fig. 2c) and the optical properties (that is, thickness and refractive index of individual layers), we can theoretically predict the normalized photoluminescence spectrum as a function of the VO_2_ state (see Methods section and Supplementary Fig. 4). Using the measured refractive indices of VO_2_ in the insulating state, as shown in Fig. 3c, the theoretical prediction (black line) matches well with the experimental spectrum without 1,064 nm laser illumination (shaded red area). For the insulating state, these calculations indicate that ∼70% of the total emission originates from MD transitions. Furthermore, as shown in Fig. 3d, when we use the measured refractive indices of VO_2_ in the metallic state, we accurately predict the experimental spectrum obtained under 1,064 nm illumination. In the metallic state, the calculations suggest that MD transitions account for a much smaller percentage of emission (∼21%) and the vast majority of emission (∼79%) originates from ED transitions.

### Modulating spontaneous emission faster than lifetime limit

We have therefore experimentally demonstrated that light emission from Er^3+^ ions around 1.5 μm can be controllably switched from MD-dominant emission to ED-dominant emission. The result of this tuning is a significant modulation of the spectral shape, intensity and polarization of light emission. But the two most important implications of using a phase-change material to modulate light emission are the following: (i) The resulting modulation is broadband: in this experiment it covers the S-, C- and L-bands of conventional fibre-optic communication. Furthermore, given the ultra-broadband tuning of VO_2_ refractive index (Supplementary Fig. 5), the modulation range can easily be extended to shorter wavelengths (down to ∼500 nm). (ii) This all-optical switching exploits modifications to the LDOS rather than pumping on and off the electronic system governing light emission (Supplementary Figs 6 and 7). There is a fundamental difference in the time-scales of these two processes. For example when Er^3+^ ions are subjected to a pulsed excitation, the time-scale of the decay process is intrinsically limited by the long lifetime of the emitters. This is clearly evidenced in Fig. 4b, where full decay of photoluminescence from the ^4^*I*_13/2_ excited state requires almost 10 ms. On the other hand, if we use the 532 nm laser as a continuous excitation source and a pulsed 1,064 nm laser to dynamically switch the VO_2_ phase, we can modulate the emission much faster than the decay time of Er^3+^.

Figure 4c presents the normalized time-resolved photoluminescence in two wavelength ranges of interest: the S-band (1,450–1,520 nm; ED-dominant, blue line) and the C-band (1,540–1,560 nm; MD-dominant, red line) when the 1,064 nm control laser is chopped by an acousto-optic modulator (AOM) at a repetition rate of 200 kHz (see Fig. 2a for a schematic of the set-up and Methods for a detailed description of the measurements). We are able to clearly modulate light emission at time-scales more than three orders of magnitude faster than the lifetime of Er^3+^ ions. (Note that without the VO_2_ layer, no modulation is observed). In addition, we observe that when the S-band ED-emission (blue line) is enhanced, the C-band MD-emission (red line) is simultaneously suppressed: a clear indication that the modulated emission is consistent with the linked ED and MD LDOS changes. Note that the observed intensity modulation ratio of 2:1 results from our use of spectrally mixed ED and MD transitions. Even when switching from strongly MD emission (72% MD) to strongly ED emission (21% MD), the resulting intensity modulation will be lower than the average rate change (for example, 72%/21%), because ED and MD emission is mixed throughout the wavelength region of interest. One could achieve greater modulation depths by leveraging further differences beyond spectral intensity, such as the differing phase and polarization symmetries of ED and MD emission^22^. For example, the 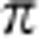-phase shift between ED and MD transitions could be used for differential phase shift keying modulation^23^.

## Discussion

We expect the fundamental limit on the speed of modulation to be significantly faster than this initial demonstration. In our experiment, the speed of switching is mainly limited by the AOM used to modulate the 1,064 nm control laser. In theory, dynamic modulation of emission through tuning the LDOS is only limited by retardation effects (that is, the time needed for light to propagate from the emitter to the reflecting layer and back). Therefore, the proposed device should allow modulation speeds approaching the time-scale of the IMT of VO_2_, which may be as fast as a few hundreds of femtoseconds^2^,^3^. The ultimate switching speed of our device could be tested in future studies by using an ultrafast laser (for example, Ti:Sapphire) to switch VO_2_ (refs 2, 4), while continuously pumping Er ions and monitoring their luminescence. Alternatively, one could electrically modulate the VO_2_ layer to eliminate the need for ultrafast lasers and, in turn, investigate device performance in a more practical operating mode.

Using phase-change media such as VO_2_ to dynamically control spontaneous emission allows for complete device integration onto a single chip. Such a monolithic and heterogeneous integration is demonstrated here for the first time. Moreover, the use of phase-change materials dramatically increases the speed of LDOS modulation by orders of magnitude over initial mechanical approaches using piezoelectrically actuated mirrors^14^. Dynamic modulation of spontaneous emission in this monolithic nanoscale device could potentially reach ultrafast speeds, up to the phase transition kinetics of VO_2_.

The presented device has a very simple geometry: it is composed of a stack of planar nanolayers. (See Supplementary Fig. 8 for demonstration of sub-lifetime modulation of Er^3+^ emission using a different multilayer stack with a silicon spacer and gold mirror.) Although the respective thicknesses are crucial for engineering the modulation amplitude, there are no restrictions on the lateral dimensions. Indeed, in this experiment, both laser beams were confined to a diffraction-limited spot. Such a device could therefore easily be integrated on a variety of structures including cavities, waveguides and light-emitting devices. While this initial demonstration focused on all-optical modulation, switching of VO_2_ could also be produced by electrically triggering the IMT^24^,^25^ (see Supplementary Note 2 for discussion of switching energy). Combined with electroluminescent devices^26^,^27^, all-electrical direct modulation of emission could be obtained. The presented concept is not limited to Er^3+^ ions and VO_2_. Indeed, the dynamic modulation of the LDOS could be extended to any phase-change material which experiences a large change in optical properties^1^,^5^,^7^,^28^ and any emitters with spectrally close ED and MD transitions. Such emitters include both lanthanide^14^,^21^ and transition-metal doped materials^29^,^30^ that are widely used as phosphors in solid-state light sources. We therefore hope that the device and concept presented here will engage both academic and industrial researchers working on optoelectronics and nanophotonics.

## Methods

### Device fabrication

A 145-nm-thick Y_2_O_3_ buffer-layer was deposited by e-beam evaporation followed by a 50-nm-thick Er^3+^:Y_2_O_3_ thin-film emitter and covered by a ∼5-nm Y_2_O_3_ layer. The sample was then annealed for 1 h at 900 °C under a flux of O_2_ (0.5 lpm) to both activate the Er^3+^ ions and crystallize the Y_2_O_3_. A TiO_2_ spacer layer was subsequently deposited by reactively sputtering a pure titanium target under a controlled O_2_/Ar ratio (5%/95%). A low-temperature anneal (500 °C, 0.5 lpm O_2_) was carried out to homogenize the TiO_2_ layer. On top of this spacer layer, VO_2_ was deposited by sputtering a V_2_O_5_ target under a partial atmosphere of O_2_/Ar (0.08 sccm O_2_ and 49.92 sccm Ar) while maintaining the substrate at 550 °C (ref. 24). Finally, a 200-nm silver layer was sputtered from a pure Ag target under an atmosphere of pure argon. All layer thicknesses, except the VO_2_ layer, were monitored *in situ* using a quartz microbalance. All refractive indices and thicknesses were measured after deposition by ellipsometry.

### Experimental set-up

The conventional photoluminescence spectrum of the sample was obtained using an inverted microscope in which an oil immersion ( × 100, 1.3 NA) objective was used for both excitation of the sample and collection of light emission. Er^3+^ ions were pumped using a 532-nm frequency-doubled Nd:YVO_4_ laser (Coherent Verdi). The laser line and emission from the sample were separated by a 665-nm dichroic mirror. The near-infrared emission was directed to an imaging spectrograph (IsoPlane SCT 320), subsequently dispersed (grating with 300 lines per mm blazed at 1.2 μm) and detected by a 2D InGaAs detector array (NIRvana, Princeton Instruments). Measured data have been corrected for the spectral and polarization dependence of the optical set-up using a calibrated quartz tungsten halogen lamp (Newport, Oriel 63355).

For energy-momentum spectroscopy, a 100-mm Bertrand lens was used to image the back focal plane of the objective and thus project the radiation pattern of emission onto the entrance slit of the spectrograph. Using a rotatable polarizer, *s*- and *p*-polarized back focal plane spectra were then obtained by imaging the energy- and momentum-resolved photoluminescence on the 2D NIR camera. The all-optical modulation experiment was performed by focusing a 1,064-nm laser on the sample through the same × 100 objective. The laser line was filtered out by a 1,064-nm dichroic mirror and a 1,064-nm long pass filter. Precise coalignment of the 532 nm and the 1,064-nm lasers was enabled by using an eyepiece camera to monitor the position of their respective foci. Spectra of Er^3+^:Y_2_O_3_ with VO_2_ in the insulating and metallic states were obtained similarly to the photoluminescence set-up described above, by simply turning on and off the 1,064 nm laser. Time-resolved photoluminescence was acquired by a time-gated fiber-coupled InGaAs/InP single-photon avalanche photodiode, and a multichannel analyser (Stanford Research System 430) was used to build a histogram of photon arrival times. For conventional lifetime measurements of Er^3+^ ions, the 532 nm laser was modulated by a mechanical chopper at a frequency of 38 Hz. Dynamic modulation of the LDOS through VO_2_ switching was carried out by chopping the 1,064 nm laser with an AOM. Two long pass filters (1,350 nm and 1,450 nm) were cascaded (total OD 10) and systematically used to block the laser line at the entrance of the single-photon avalanche photodiode, and additional bandpass filters were used to measure specific wavelength ranges.

### Quantification of intrinsic emission rates

To determine the intrinsic ED and MD emission rates, we measured the energy-momentum spectra of the Er^3+^:Y_2_O_3_ emitter layer on quartz in a region without the TiO_2_/VO_2_/Ag overcoat layers (Supplementary Fig. 1). With this technique, we measure the distribution of light emission from Er^3+^ as a function of wavelength 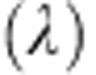 and in-plane momentum (*k*_||_). Using the procedure first described in Taminiau *et al.*^22^, experimental energy-momentum cross-sections in this three-layer system were fit to:


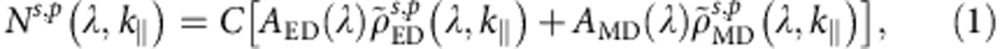


where *C* is an overall scaling factor to account for experimental parameters that influence the number of measured counts but do not affect the radiation pattern. 
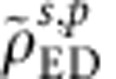
 and 
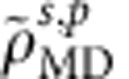
 are the normalized LDOS for a three-layer system calculated using equations in the Supplementary Information (S11–S14) of ref. 22. (Note that the three-layer LDOS expressions may be readily derived from the five-layer system expressions provided in Supplementary Note 1 by setting *n*_0_=*n*_1_=*n*_2_=1). Using equation (1), we can fit the energy-momentum spectra to extract the spectrally resolved Einstein *A* coefficients (*A*_ED_ and *A*_MD_). These coefficients, shown in Fig. 2c, represent the relative ED and MD intrinsic radiative rates at each wavelength, and are proportional to the intrinsic rates that one would expect in a homogeneous medium.

### Theoretical calculation of modified emission spectra

To predict how the Er^3+^ emission spectrum will be modified by the VO_2_ phase-change, we use the measured intrinsic emission rates, 
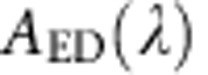
 and 
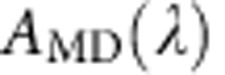
, inferred from the three-layer, emitter-on-substrate system together with the theoretically calculated LDOS for the five-layer device structure (described in Supplementary Note 1). The product of the intrinsic emission rate with the appropriate integrated LDOS yields the radiative decay rate for electric and magnetic transitions:


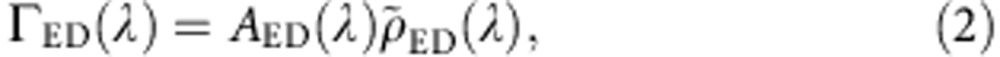



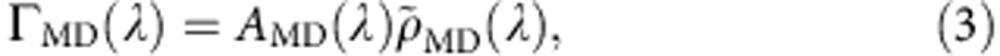


where the LDOS is integrated in momentum over the range of values collected by the numerical aperture (NA) of our imaging system:


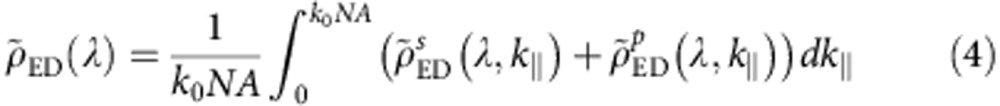


and


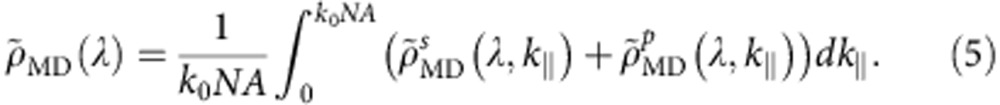


Note that in equations (4 and 5) we integrate over a line in momentum-space, which is consistent with the experimental set-up where the spectrum is collected after a Bertrand lens through a narrow slit spectrometer.

The intensity of emitted light is proportional to the total emission rate, 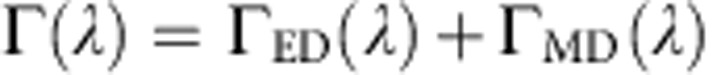. Therefore, the theoretical normalized intensity can be calculated as follows:


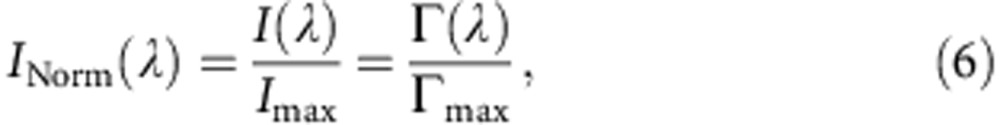


where *I*_max_ and Γ_max_ denote the maximum values of the light intensity and the total emission rate near 1.5 μm, respectively. Using equations (2, 3, 4, 5, 6), we can calculate the predicted emission spectrum of Er^3+^ for both phases of VO_2_. As can be seen in Fig. 3c,d, there is an excellent agreement between the predicted and measured spectra. For reference, Supplementary Fig. 4 shows the calculated spectra (black line) decomposed into ED (shaded blue area) and MD (shaded red area) contributions.

### Simulation of branching ratios

To quantify the fraction of light emission that originates from MD or ED transitions, we define branching ratios (
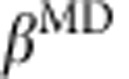
 and 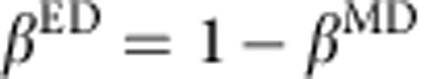) as a function of the TiO_2_ spacer layer thickness 
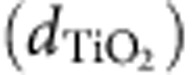
 and VO_2_ layer thickness 
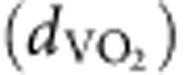
:


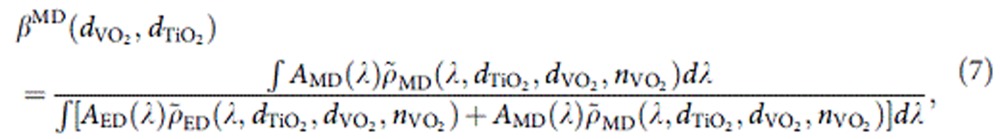


where 
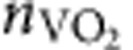
 is the phase-dependent refractive index of VO_2_. In our calculations, we use wavelength-dependent data for VO_2_ at 25 and 95 °C measured by Jianing Sun of the J.A. Woollam Company to model the insulating and metallic phases, respectively (Supplementary Fig. 5). For the complex refractive index of the silver layer, we use the Lorentz–Drude model in Rakić *et al.*^31^.

## Additional information

**How to cite this article:** Cueff, S. *et al.* Dynamic control of light emission faster than the lifetime limit using VO_2_ phase-change. *Nat. Commun.* 6:8636 doi: 10.1038/ncomms9636 (2015).

## Supplementary Material

Supplementary Information Supplementary Figures 1-8, Supplementary Notes 1-2 and Supplementary References

## References

[b1] Loke D. *et al.* Breaking the speed limits of phase-change memory. Science 336, 1566–1569 (2012). 22723419 10.1126/science.1221561

[b2] Cavalleri A. *et al.* Femtosecond structural dynamics in VO_2_ during an ultrafast solid-solid phase transition. Phys. Rev. Lett. 87, 237401 (2001). 11736474 10.1103/PhysRevLett.87.237401

[b3] Appavoo K. *et al.* Ultrafast phase transition via catastrophic phonon collapse driven by plasmonic hot-electron injection. Nano Lett. 14, 1127–1133 (2014). 24484272 10.1021/nl4044828

[b4] Lysenko S., Rúa A., Vikhnin V., Fernández F. & Liu H. Insulator-to-metal phase transition and recovery processes in VO_2_ thin films after femtosecond laser excitation. Phys. Rev. B 76, 035104 (2007).

[b5] Hosseini P., Wright C. D. & Bhaskaran H. An optoelectronic framework enabled by low-dimensional phase-change films. Nature 511, 206–211 (2014). 25008527 10.1038/nature13487

[b6] Kats M. A. *et al.* Ultra-thin perfect absorber employing a tunable phase change material. Appl. Phys. Lett. 101, 221101 (2012).

[b7] Samson Z. *et al.* Metamaterial electro-optic switch of nanoscale thickness. Appl. Phys. Lett. 96, 143105 (2010).

[b8] Ryckman J. D., Hallman K. A., Marvel R. E., Haglund R. F. & Weiss S. M. Ultra-compact silicon photonic devices reconfigured by an optically induced semiconductor-to-metal transition. Opt. Express 21, 10753–10763 (2013). 23669932 10.1364/OE.21.010753

[b9] Curto A. G. *et al.* Unidirectional emission of a quantum dot coupled to a nanoantenna. Science 329, 930–933 (2010). 20724630 10.1126/science.1191922

[b10] Strauf S. *et al.* High-frequency single-photon source with polarization control. Nat. Photon. 1, 704–708 (2007).

[b11] Noda S., Yokoyama M., Imada M., Chutinan A. & Mochizuki M. Polarization mode control of two-dimensional photonic crystal laser by unit cell structure design. Science 293, 1123–1125 (2001). 11498586 10.1126/science.1061738

[b12] Karaveli S. & Zia R. Spectral tuning by selective enhancement of electric and magnetic dipole emission. Phys. Rev. Lett. 106, 193004 (2011). 21668150 10.1103/PhysRevLett.106.193004

[b13] Li D. *et al.* Quantifying and controlling the magnetic dipole contribution to 1.5-*μ*m light emission in erbium-doped yttrium oxide. Phys. Rev. B 89, 161409 (2014).

[b14] Karaveli S., Weinstein A. J. & Zia R. Direct modulation of lanthanide emission at sub-lifetime scales. Nano Lett. 13, 2264–2269 (2013). 23597062 10.1021/nl400883r

[b15] Matsui Y., Murai H., Arahira S., Kutsuzawa S. & Ogawa Y. 30-GHz bandwidth 1.55-*μ*m strain-compensated InGaAlAs-InGaAsP MQW laser. Photon. Technol. Lett. IEEE 9, 25–27 (1997).

[b16] Kjebon O. *et al.* 30 GHz direct modulation bandwidth in detuned loaded InGaAsP DBR lasers at 1.55 *μ*m wavelength. Electron. Lett. 33, 488–489 (1997).

[b17] Mohrdiek S. *et al.* 10-Gb/s standard fiber transmission using directly modulated 1.55-*μ*m quantum-well DFB lasers. Photon. Technol. Lett. IEEE 7, 1357–1359 (1995).

[b18] Liu A. *et al.* A high-speed silicon optical modulator based on a metal-oxide-semiconductor capacitor. Nature 427, 615–618 (2004). 14961115 10.1038/nature02310

[b19] Xu Q., Schmidt B., Pradhan S. & Lipson M. Micrometre-scale silicon electro-optic modulator. Nature 435, 325–327 (2005). 15902253 10.1038/nature03569

[b20] Reed G. T., Mashanovich G., Gardes F. & Thomson D. Silicon optical modulators. Nat. Photon. 4, 518–526 (2010).

[b21] Dodson C. M. & Zia R. Magnetic dipole and electric quadrupole transitions in the trivalent lanthanide series: calculated emission rates and oscillator strengths. Phys. Rev. B 86, 125102 (2012).

[b22] Taminiau T. H., Karaveli S., van Hulst N. F. & Zia R. Quantifying the magnetic nature of light emission. Nat. Commun. 3, 979 (2012). 22864572 10.1038/ncomms1984

[b23] Winzer P. J. & Essiambre R.-J. Advanced optical modulation formats. Proc. IEEE 94, 952–985 (2006).

[b24] Zhou Y. *et al.* Voltage-triggered ultrafast phase transition in vanadium dioxide switches. IEEE Electron Device Lett. 34, 220–222 (2013).

[b25] Joushaghani A. *et al.* Electronic and thermal effects in the insulator-metal phase transition in VO_2_ nano-gap junctions. Appl. Phys. Lett. 105, 231904 (2014).

[b26] Cueff S. *et al.* Structural factors impacting carrier transport and electroluminescence from Si nanocluster-sensitized Er ions. Opt. Express 20, 22490–22502 (2012). 23037398 10.1364/OE.20.022490

[b27] Cueff S. *et al.* Electroluminescence efficiencies of erbium in silicon-based hosts. Appl. Phys. Lett. 103, 191109 (2013).

[b28] Shi J., Zhou Y. & Ramanathan S. Colossal resistance switching and band gap modulation in a perovskite nickelate by electron doping. Nat. Commun. 5, 4860 (2014). 25181992 10.1038/ncomms5860

[b29] Karaveli S., Wang S., Xiao G. & Zia R. Time-resolved energy-momentum spectroscopy of electric and magnetic dipole transitions in Cr^3+^: MgO. ACS Nano 7, 7165–7172 (2013). 23879390 10.1021/nn402568d

[b30] Karaveli S., Li D. & Zia R. Probing the electromagnetic local density of states with a strongly mixed electric and magnetic dipole emitter. Preprint at http://arxiv.org/abs/1311.0516 (2013).

[b31] Rakić A. D., Djurišic A. B., Elazar J. M. & Majewski M. L. Optical properties of metallic films for vertical-cavity optoelectronic devices. Appl. Opt. 37, 5271–5283 (1998). 18286006 10.1364/ao.37.005271

