# Supplementary material for: Dynamic control of light emission faster than the lifetime limit using VO2 phase-change
Source: Nat Commun. 2015 Oct 22;6:8636. doi: 10.1038/ncomms9636 (PMC4639901; doi:10.1038/ncomms9636)
Supplement: Supplementary Information — Supplementary Figures 1-8, Supplementary Notes 1-2 and Supplementary References [file ncomms9636-s1.pdf]

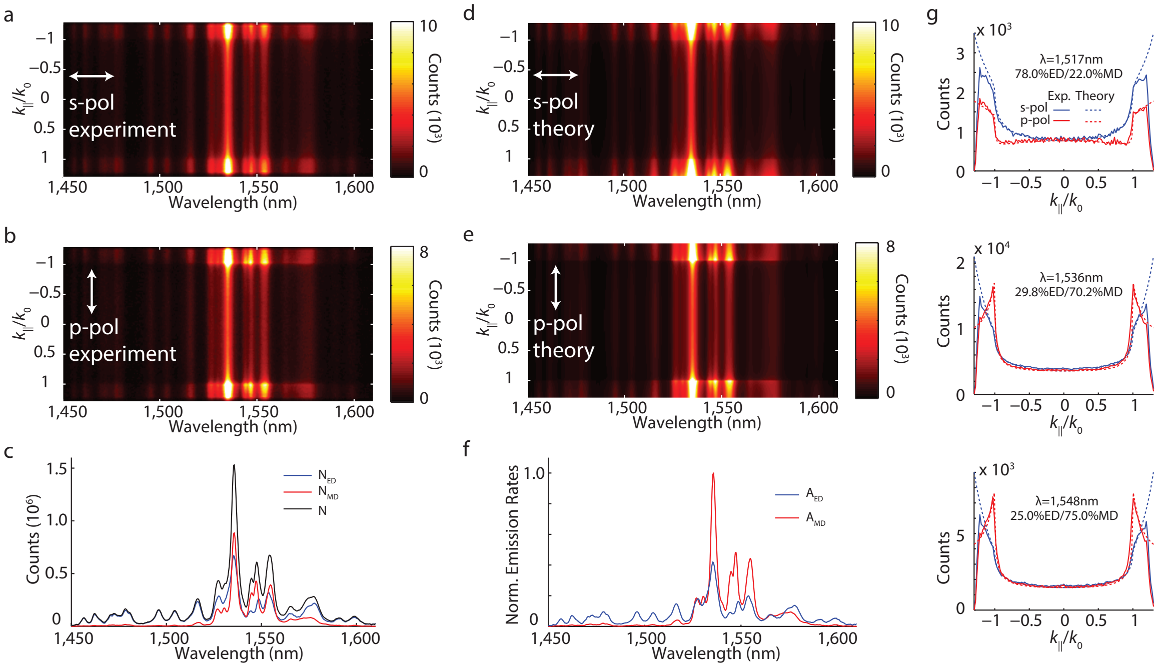

Supplementary Figure 1: **Quantifying ED and MD contributions to Er^{3+} emission around 1.5 μm using energy-momentum spectroscopy.** (**a,b**) Experimental energy-momentum spectra for s- and p-polarization. (**c**) ED and MD contributions to the total light emission. (**d,e**) Theoretical fits of energy-momentum spectra for s- and p-polarization. (**f**) Spectrally-resolved intrinsic emission rates for ED emission (blue) and MD emission (red), obtained by fitting the experimentally measured momentum cross-sections. (**g**) Experimentally measured momentum cross sections at three different wavelengths along with associated fits.

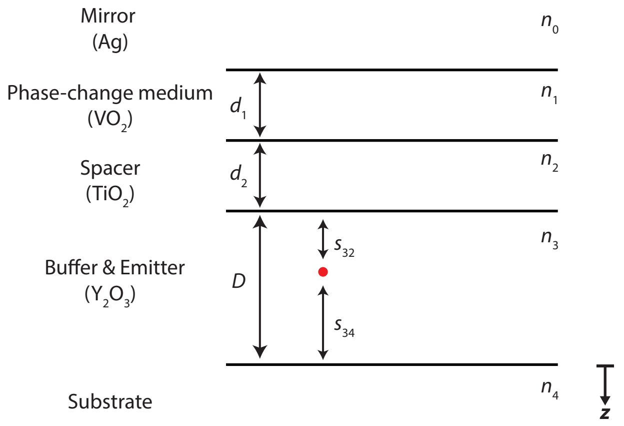

Supplementary Figure 2: **Schematic of the five-layer system.** The optical response of the multilayer structure proposed and fabricated in this study was modeled using the five-layer system illustrated here. It is composed (from bottom to top) of: a quartz substrate with refractive index n_4; a combined buffer and emitter Y_2O_3 layer with a thickness D and refractive index n_3; a TiO_2 spacer with a thickness d_2 and refractive index n_2; a VO_2 layer of thickness d_1 and refractive index n_1; and topped by a silver mirror with a refractive index n_0. The emitting point dipole (depicted in red) is located at a distance s_{34} from the substrate and s_{32} from the TiO_2 spacer. We model the system assuming emitters at the center of the doped region (Er^{3+}:Y_2O_3), and use asymmetric s_{32} and s_{34} distances (i.e. s_{32}=30 nm and s_{34}=170 nm) to model the buffer and emitter layers as an optically continuous Y_2O_3 region.

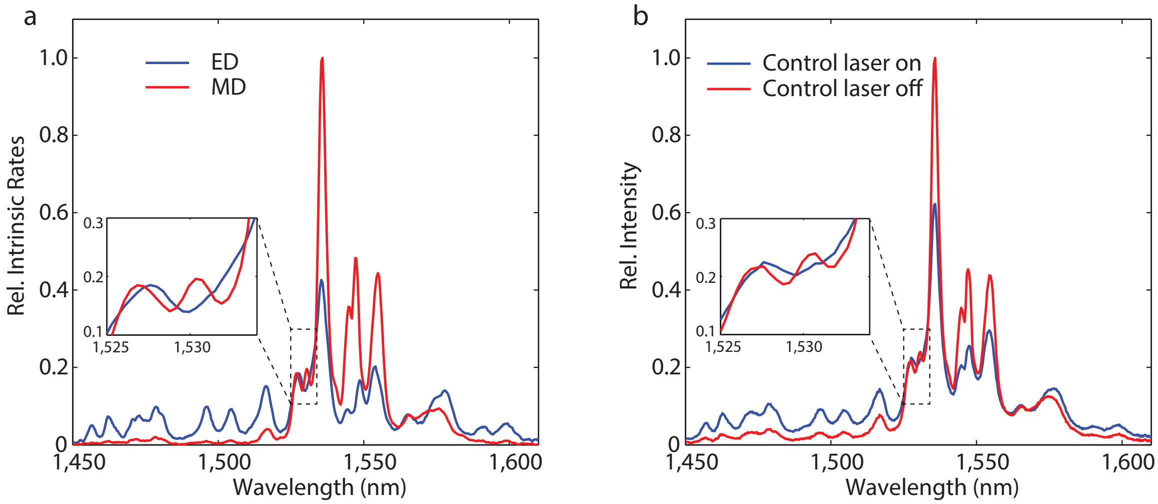

Supplementary Figure 3: **Comparison of intrinsic emission rates and measured spectra.** (**a**) Normalized intrinsic ED (blue) and MD (red) emission rates extracted from energy-momentum spectroscopy. (**b**) Normalized far-field spectra measured when VO_2 is in its insulating state (red) and switched to its metallic state (blue) by a 1,064 nm control laser. Side-by-side comparison of (**a**) and (**b**) clearly reveals that when the control laser is off, mainly MD emission is observed, whereas dominant ED emission is obtained when control laser is on. The zoomed insets highlight spectra in the wavelength range 1,525-1,534 nm. The inset of (**a**) shows a trough at $\sim$1,530 nm for the intrinsic ED rate, while for intrinsic MD rate there is a peak. Interestingly, these fine features are well reproduced experimentally in the inset of (**b**) further indicating a clear switch from ED to MD emission.

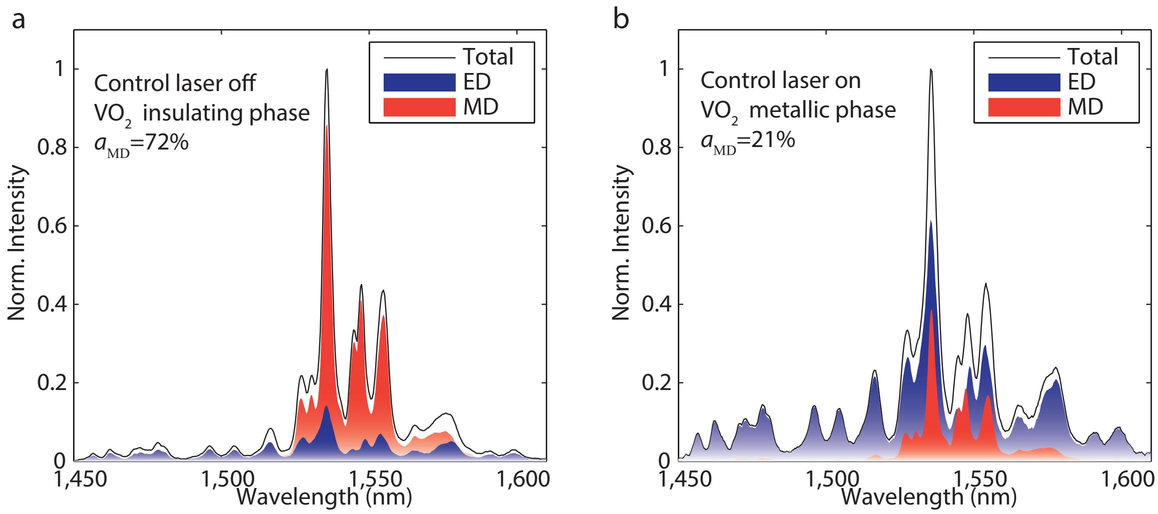

Supplementary Figure 4: **Theoretical calculation of ED and MD spectra for VO_2 in (a) insulating phase (control laser off) and (b) metallic phase (control laser on)**. The shaded red area denotes the MD contribution while the shaded blue area represents the ED contribution. Their summation is plotted by the solid black lines, which are included in Fig. 3c,d of the main text.

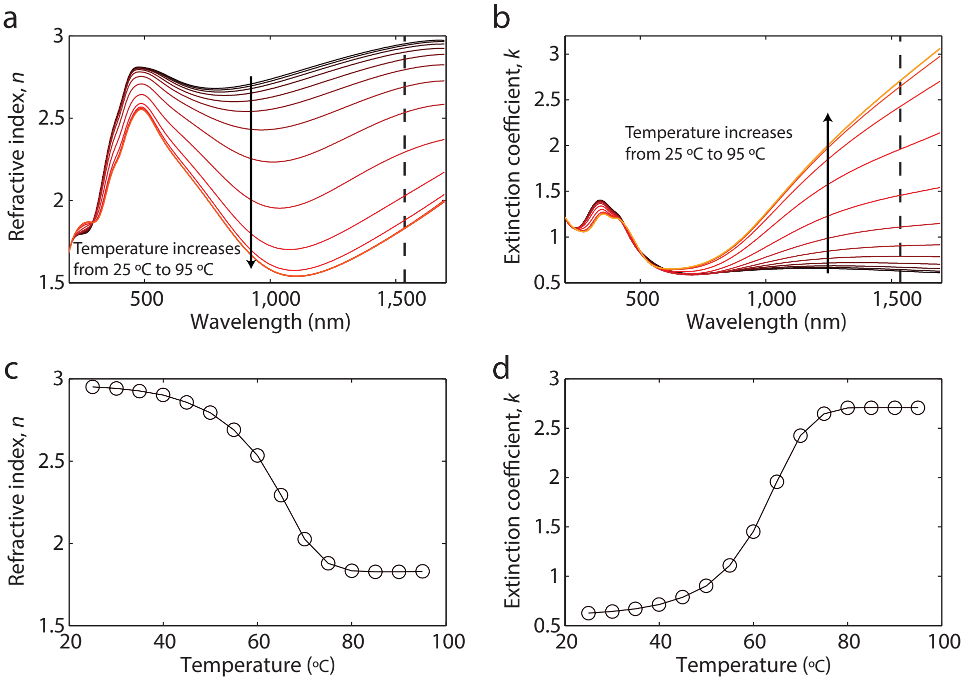

Supplementary Figure 5: **Refractive index of VO_2.** The temperature-dependent complex refractive index and extinction coefficient of VO_2 deposited on a silicon substrate were measured by Jianing Sun of the J.A. Woollam Company with a M-2000DI spectroscopic ellipsometer, and are included here for completeness. (**a,b**) are spectroscopic ellipsometry measurements of the temperature-dependent complex refractive index of VO_2 from 25°C to 95°C with a step 5°C in the range 200 - 1,700 nm. (**c,d**) are the refractive index n and extinction coefficient k respectively at 1,535 nm as labeled by the dashed black lines in (**a**) and (**b**). Panels (**c**) and (**d**) show how the refractive index at 1,535 nm (near the range of interest) approaches constant values at the two extremes of this temperature range. Therefore, in our calculations, we use the measured wavelength-dependent data for VO_2 at 25°C and 95°C to model the insulating and metallic phases, respectively.

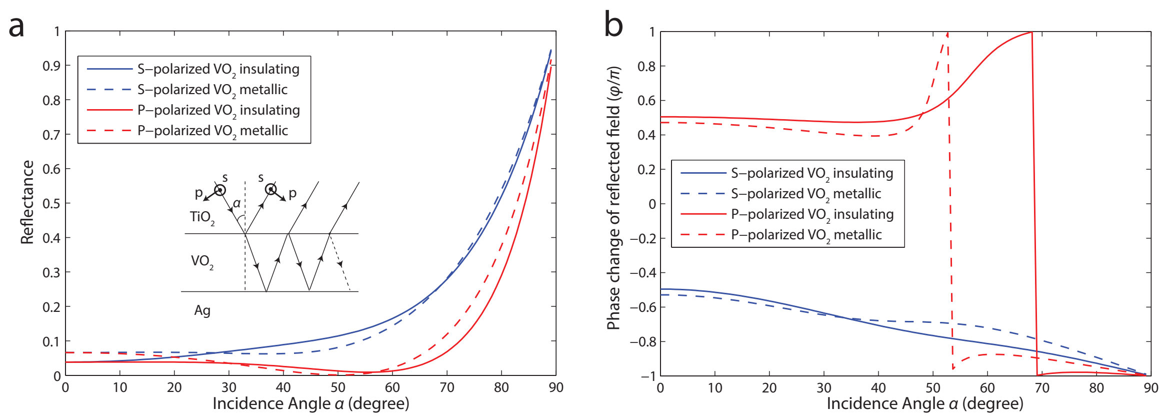

Supplementary Figure 6: **Calculated effect of VO_2 phase-change on the reflection of the 532 nm pump laser.** **(a,b)** Reflectance and phase change from the multilayer structure composed of TiO_2, VO_2 (110-nm-thick), and Ag for both s-(blue) and p-(red) polarization in insulating (solid) and metallic (dashed) phases. In both its metallic and insulating phases, VO_2 exhibits considerable absorption at the excitation wavelength of 532 nm, and therefore, VO_2 phase-change does not induce a significant change in pump intensity at the emitter layer. To confirm this, (**a**) shows the reflectance for 532 nm light incident upon our TiO_2/VO_2/Ag multilayer structure. Within the half-angle of our 1.3 NA objective (i.e., $\sin^{-1}(1.3/2.48) = 31.6°$), the reflectance for both metallic and insulating phases remains below 10%. There is also no appreciable change in the reflected phase, as shown in (**b**). This suggests that VO_2 phase-change should not induce pump-based modulation of erbium emitters. This is confirmed by the fact that comparable intensities of Er^{3+} emission are observed for both VO_2 phases, as shown in Supplementary Fig. 7.

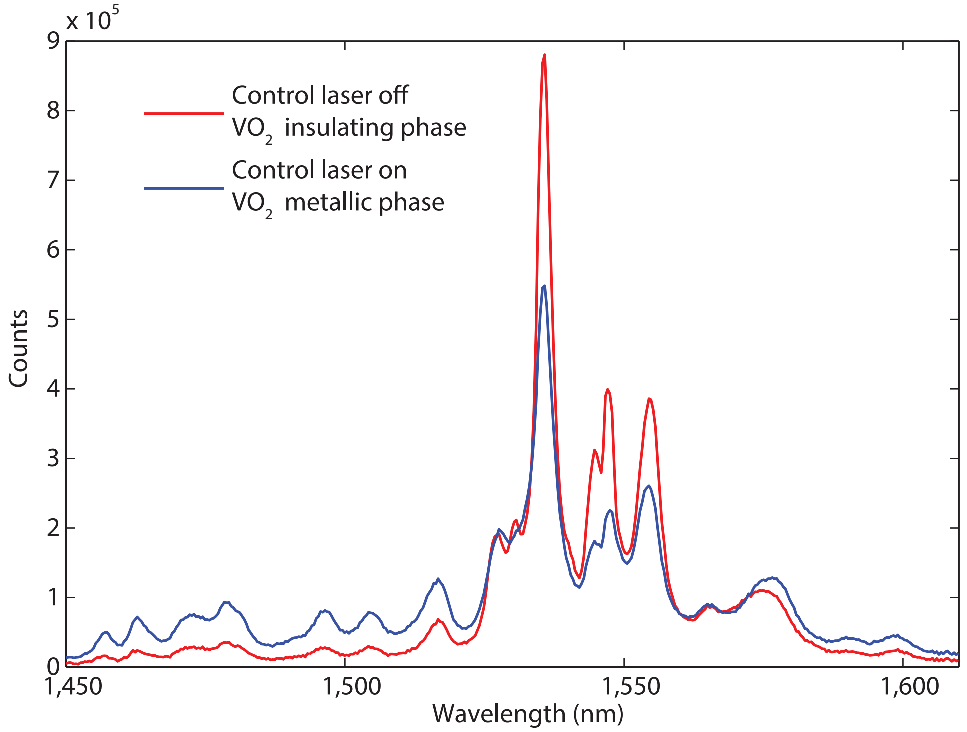

Supplementary Figure 7: **Direct comparison of as-collected Er^{3+}:Y_2O_3 photoluminescence for insulating and metallic phases.** Note that the total integrated counts for the two phases are within 7% of each other. The comparable intensities of Er^{3+} emission for both phases help confirm that VO_2 phase-change should not induce pump-based modulation of erbium emitters, as suggested by the theoretical results shown in Supplementary Fig. 6.

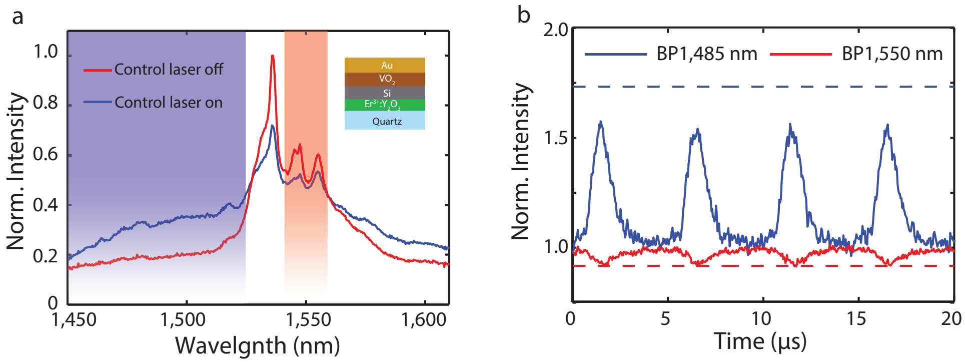

Supplementary Figure 8: **Sub-lifetime modulation of Er^{3+} light emission for a sample with silicon spacer and gold mirror.** (**a**) Experimental spectra for Er^{3+} ions when VO_2 is in the insulating state (red) and in the metallic state (blue). Here, the absence of a buffer below the emitter layer results in poor crystallization, which leads to a broader emission spectrum with less well-defined emission peaks. The sample was fabricated as follows: a 50-nm-thick Er^{3+}:Y_2O_3 thin-film emitter layer was deposited on a quartz substrate by e-beam evaporation. The sample was then annealed for 1h at 900°C under a flux of O_2 (0.5 lpm) to both activate the Er^{3+} ions and crystallize the Y_2O_3. A 55-nm-thick silicon spacer layer was subsequently deposited by sputtering a pure silicon target under Ar flow. On top of this spacer layer, VO_2 was deposited by sputtering a V_2O_5 target in an O_2/Ar gas mixture (0.08 sccm O_2 and 49.92 sccm Ar) while maintaining the substrate at 550 °C. Finally, a 5 nm adhesion layer of titanium and a 200 nm gold layer were deposited by e-beam evaporation. (**b**) Time-resolved normalized photoluminescence (under CW 532 nm excitation) while VO_2 phase is switched using a pulsed 1,064 nm laser at a 200 kHz repetition rate. Blue and red solid lines represent two different spectral ranges of the bandpass filters (1,450 nm - 1,520 nm and 1,538 - 1,562 nm, respectively). The dashed lines show the theoretical maximum modulation inferred from the experimental spectra in (**a**).

Supplementary Note 1. Normalized LDOS for five-layer structure

A sketch of the five-layer device geometry for the optical modulation experiments is shown in Supplementary Fig. 2. The normalized electric and magnetic LDOS of such a structure are calculated below in Supplementary equations (1)-(4):

$$\tilde{\rho}^{\mathrm{p}}_{\mathrm{ED}}\left(\lambda, k_{\parallel}\right) = \frac{k_4 n_4}{8\pi k_4^2 k_{\mathrm{z}4} n_3}\left|\frac{t^{\mathrm{p}}_{43} e^{ik_{\mathrm{z}3}s_{34}}}{1 - R^{\mathrm{p}}_{32} r^{\mathrm{p}}_{34} e^{2ik_{\mathrm{z}3}(s_{34}+s_{32})}}\right|^2 \left(\left|\frac{k_{\mathrm{z}3}}{k_3}\left(1 - R^{\mathrm{p}}_{32} e^{2ik_{\mathrm{z}3}s_{32}}\right)\right|^2 + \left|\frac{k_{\parallel}}{k_3}\left(1 + R^{\mathrm{p}}_{32} e^{2ik_{\mathrm{z}3}s_{32}}\right)\right|^2\right), \tag{1}$$

$$\tilde{\rho}^{\mathrm{s}}_{\mathrm{ED}}\left(\lambda, k_{\parallel}\right) = \frac{k_4 n_4}{8\pi k_4^2 k_{\mathrm{z}4} n_3}\left|\frac{t^{\mathrm{s}}_{43} e^{ik_{\mathrm{z}3}s_{34}}}{1 - R^{\mathrm{s}}_{32} r^{\mathrm{s}}_{34} e^{2ik_{\mathrm{z}3}(s_{34}+s_{32})}}\left(1 + R^{\mathrm{s}}_{32} e^{2ik_{\mathrm{z}3}s_{32}}\right)\right|^2, \tag{2}$$

$$\tilde{\rho}^{\mathrm{p}}_{\mathrm{MD}}\left(\lambda, k_{\parallel}\right) = \frac{k_4 n_4}{8\pi k_4^2 k_{\mathrm{z}4} n_3}\left|\frac{t^{\mathrm{p}}_{43} e^{ik_{\mathrm{z}3}s_{34}}}{1 - R^{\mathrm{p}}_{32} r^{\mathrm{p}}_{34} e^{2ik_{\mathrm{z}3}(s_{34}+s_{32})}}\left(1 + R^{\mathrm{p}}_{32} e^{2ik_{\mathrm{z}3}s_{32}}\right)\right|^2, \tag{3}$$

$$\tilde{\rho}^{\mathrm{s}}_{\mathrm{MD}}\left(\lambda, k_{\parallel}\right) = \frac{k_4 n_4}{8\pi k_4^2 k_{\mathrm{z}4} n_3}\left|\frac{t^{\mathrm{s}}_{43} e^{ik_{\mathrm{z}3}s_{34}}}{1 - R^{\mathrm{s}}_{32} r^{\mathrm{s}}_{34} e^{2ik_{\mathrm{z}3}(s_{34}+s_{32})}}\right|^2 \left(\left|\frac{k_{\mathrm{z}3}}{k_3}\left(1 - R^{\mathrm{s}}_{32} e^{2ik_{\mathrm{z}3}s_{32}}\right)\right|^2 + \left|\frac{k_{\parallel}}{k_3}\left(1 + R^{\mathrm{s}}_{32} e^{2ik_{\mathrm{z}3}s_{32}}\right)\right|^2\right), \tag{4}$$

where

$$r^{\mathrm{s}}_{ij} = \frac{k_{zi} - k_{zj}}{k_{zi} + k_{zj}},\ r^{\mathrm{p}}_{ij} = \frac{k_{\mathrm{z}i} n_j^2 - k_{\mathrm{z}j} n_i^2}{k_{\mathrm{z}i} n_j^2 + k_{\mathrm{z}j} n_i^2},\ t^{\mathrm{s}}_{ij} = \frac{2k_{\mathrm{z}i}}{k_{\mathrm{z}i} + k_{\mathrm{z}j}},\ t^{\mathrm{p}}_{ij} = \frac{2k_{\mathrm{z}i} n_j^2}{k_{\mathrm{z}i} n_j^2 + k_{\mathrm{z}j} n_i^2}\frac{n_i}{n_j} \tag{5}$$

$$R^{s,p}_{32} = \frac{r^{s,p}_{32} + R^{s,p}_{21} e^{2ik_{\mathrm{z}2}d_2}}{1 + r^{s,p}_{32} R^{s,p}_{21} e^{2ik_{\mathrm{z}2}d_2}},\ R^{s,p}_{21} = \frac{r^{s,p}_{21} + r^{s,p}_{10} e^{2ik_{\mathrm{z}1}d_1}}{1 + r^{s,p}_{21} r^{s,p}_{10} e^{2ik_{\mathrm{z}1}d_1}}. \tag{6}$$

Supplementary Note 2. Switching energy

The switching energy of the thermal phase transition gives an upper limit for electrically and optically triggered transitions, as the thermal transition proceeds all the way to the rutile phase. The volumetric enthalpy of switching VO_2 from monoclinic to the rutile phase is $\sim 3.6\times 10^8$ J m^{-3} ([1] and references therein), based on its heat capacity. Assuming a 1 μm^2 device area and 100 nm thickness, the switching energy is 36 pJ per bit.

From Ryckman *et al.* [2], the fluence needed to optically switch VO_2 is ~ 1 mJ cm^{-2} which, for a diffraction-limited spot, gives a switching energy in the 10 pJ per bit range. This energy is comparable to reports on Mach-Zehnder modulators [3,4] or ring resonators [5].

Electrical switching of VO_2 in planar devices typically requires a $\sim 10^5$ V cm^{-1} electric field and $\sim 10^4$ A cm^{-2} current density [6–10]. Assuming an upper bound of 1 μm^2 for the area of the device and a VO_2 thickness of 100 nm, we need ~ 100 μW to switch the nanolayer. Applying a 1 ns voltage pulse, we obtain a switching energy of 100 fJ per bit.

Importantly, for both optical and electrical switching, the switching energy can be further reduced by decreasing the thickness and/or the area of VO_2. This could be done by patterning the mirror in subwavelength features containing nano-inclusions of VO_2. Further decrease of the energy per bit could be obtained by shortening the pulse duration of the switching signal.

Supplementary References

1. Zhou, Y. & Ramanathan, S. Mott memory and neuromorphic devices. *Proc. IEEE* **103**, 1289–1310 (2015).

2. Ryckman, J. D., Hallman, K. A., Marvel, R. E., Haglund, R. F. & Weiss, S. M. Ultra-compact silicon photonic devices reconfigured by an optically induced semiconductor-to-metal transition. *Opt. Express* **21**, 10753–10763 (2013).

3. Liao, L. *et al.* High speed silicon Mach-Zehnder modulator. *Opt. Express* **13**, 3129–3135 (2005).

4. Green, W. M., Rooks, M. J., Sekaric, L. & Vlasov, Y. A. Ultra-compact, low RF power, 10 Gb/s silicon Mach-Zehnder modulator. *Opt. Express* **15**, 17106–17113 (2007).

5. Xu, Q., Manipatruni, S., Schmidt, B., Shakya, J. & Lipson, M. 12.5 Gbit/s carrier-injection-based silicon micro-ring silicon modulators. *Opt. Express* **15**, 430–436 (2007).

6. Ko, C. & Ramanathan, S. Observation of electric field-assisted phase transition in thin film vanadium oxide in a metal-oxide-semiconductor device geometry. *Appl. Phys. Lett.* **93**, 252101 (2008).

7. Leroy, J. *et al.* High-speed metal-insulator transition in vanadium dioxide films induced by an electrical pulsed voltage over nano-gap electrodes. *Appl. Phys. Lett.* **100**, 213507 (2012).

8. Mun, B. S. *et al.* Role of joule heating effect and bulk-surface phases in voltage-driven metal-insulator transition in VO_2 crystal. *Appl. Phys. Lett.* **103**, 061902 (2013).

9. Joushaghani, A. *et al.* Voltage-controlled switching and thermal effects in VO_2 nano-gap junctions. *Appl. Phys. Lett.* **104**, 221904 (2014).

10. Joushaghani, A. *et al.* Electronic and thermal effects in the insulator-metal phase transition in VO_2 nano-gap junctions. *Appl. Phys. Lett.* **105**, 231904 (2014).
